# Risk Factors of Chemotherapy-Induced Thrombocytopenia After Oxaliplatin-Containing Chemotherapy for Gastrointestinal Malignancies

**DOI:** 10.1007/s12029-024-01059-x

**Published:** 2024-05-07

**Authors:** Ju Li, Wanqing Wang, Kaipeng Jiang, Jiuwei Cui, Chang Wang, Tingting Liang, Yizhuo Wang, Shuhan Liu, Wenshuo Zhou

**Affiliations:** The First Hospital of Jilin University, Changchun, Jilin, China

**Keywords:** Cancer, Thrombocytopenia, Chemotherapy, Risk factors

## Abstract

**Purpose:**

Thrombocytopenia is among the most common chemotherapy-related hematologic toxicities. We aim to determine the predictors of oxaliplatin chemotherapy-induced thrombocytopenia in patients with gastrointestinal tumors to guide the clinic.

**Methods:**

Clinical data of 750 patients with a malignant gastrointestinal tumor were included as the primary cohort. Basic clinical data, serological indices, and anthropometric indices of these patients were collected. According to the presence or absence of CIT, univariate analysis was performed to identify significant factors for multivariate analysis. In R language software, nomogram was constructed based on the results of multi-factor analysis, and the calibration curve and ROC curve were drawn.

**Results:**

Univariate analysis identified 17 factors as closely related to CIT occurrence, namely age, lymph node metastasis (N) stage, metastasis (M) stage, lung metastasis, other site metastasis, chemotherapy regimen, course of treatment, total dose of oxaliplatin, AST, albumin, neutrophils, monocytes, baseline platelets, transferrin, natural killer (NK) cell, phase angle, and SMI (*P* < 0.10). The binary logistic multivariate regression analysis revealed five independent risk factors for developing CIT (*P* < 0.05), including the M stage, total dose of oxaliplatin, albumin, baseline thrombocyte count, and NK cell. Based on the results of multivariate logistic regression analysis, R software was used to establish a nomogram model. The calibration curve shows that the combined predictor has good consistency. The area under the ROC curve was 0.877 and the best cut-off value was 0.3579613 (sensitivity, 78.9%; specificity, 81.8%), which showed the better prediction efficiency.

**Conclusion:**

The total dose of oxaliplatin, M stage, albumin, baseline platelet count, and NK cell was independent risk factors for CIT. The sequentially constructed histogram model had a good predictive effect on the risk of thrombocytopenia caused by oxaliplatin chemotherapy in patients with gastrointestinal malignancies.

## Background

Cancer is a major threat to human health, and there are ~ 19.3 million individuals diagnosed with cancer and 1 million cancer-related deaths per year globally [1]. Despite the rapid development of new anti-cancer treatments in recent years, such as immunotherapy and molecular targeted therapy, systemic chemotherapy remains the preferred clinical treatment for digestive system malignancies. The oxaliplatin-containing chemotherapy regimens S-1 plus oxaliplatin (SOX), oxaliplatin + capecitabine (XELOX), and oxaliplatin + leucovorin + fluorouracil (FOLFOX) are still among the primary treatment options for gastrointestinal tumors. In particular, for gastrointestinal tract tumors, these are still auxiliary and first and second lines of treatment, making them difficult to avoid.

Anti-tumor chemotherapy drugs exert an inhibitory effect on bone marrow megakaryocytes, which leads to chemotherapy-induced thrombocytopenia (CIT), represented by peripheral blood platelet counts below 100 × 10^9^/L [2]. Among patients with solid tumors, CIT is the most common in non-small cell lung cancer (25%), ovarian cancer (24%), and colorectal cancer (CRC; 18%) [3]. A retrospective study on CIT in the United States revealed an overall incidence rate of 9.7%, with the gemcitabine regimen having the highest incidence rate (13.5%), followed by platinum-based chemotherapy regimen (13.2%). Meanwhile, the incidence of CIT caused by oxaliplatin, a commonly used chemotherapy drug in patients with gastrointestinal cancer, was reportedly 11.4% [4]. Similarly, in a retrospective, hospital-based Dutch study comprising 614 patients with solid tumors who underwent chemotherapy, thrombocytopenia occurred in 22% patients. In this study, among patients treated with a monotherapy, the greatest risk of CIT development was noted in those receiving carboplatin monotherapy (82%) or oxaliplatin monotherapy (50%), and among those receiving combination therapies, the greatest risk was in patients given gemcitabine (64%), paclitaxel (59%), carboplatin (58%), or oxaliplatin (36%) as a part of the combination [5]. In an observational study, among the 15,521 patients with solid tumors, 13% developed thrombocytopenia within 3 months, 4% of patients had grade 3, and 2% of patients had grade 4 thrombocytopenia. Among the 2537 patients with hematological malignancies, 28% developed thrombocytopenia, 16% had grade 3 thrombocytopenia, and 12% had grade 4 thrombocytopenia [6]. Unfortunately, this study did not mention details about the treatment of CIT.

Considering relevant literature and clinical practice findings, platinum-based chemotherapy was found to be associated with increased risk of thrombocytopenia. A single-center retrospective review including > 250 adult patients given first-line or second-line chemotherapy regimens for the most common solid tumors was conducted in the UK. In this study, chemotherapy regimens resulted in thrombocytopenia (platelet count threshold used in this study, < 75 × 10^9^/L) in > 10% of the cases; it also caused other complications in several cases, such as delayed infusion (39%), chemotherapy dose reduction (36%), increased bleeding risk (32%), platelet transfusions (29%), and/or chemotherapy discontinuation (7%) [7].

In recent years, increasing researchers have focused their attention on exploring the influencing factors and secondary prevention of CIT, early identification of high-risk groups for CIT, and providing individualized prevention and treatment plans for these patients. However, there are few studies on risk factors for thrombocytopenia due to oxaliplatin-containing use for gastrointestinal malignancies. One study has pointed out that age, body mass index (BMI), liver metastases, baseline neutrophil count, and baseline platelet count are independent risk factors for CIT. In another study, Razzaghdoust et al. presented a predictive model based on a single laboratory variable of high ferritin, an estimated glomerular filtration rate of < 60 mL/min/1.73 m^2^, and a BMI of < 23 kg/m^2^ for CIT in patients with solid tumors or lymphoma [8]. In view of the limited variables, small sample size, and wide range of tumor types and chemotherapy regimens, the above risk factors or predictive models have not been generalized and applied clinically. Therefore, timely detection of the risk factors for CIT and taking corresponding measures to avoid its occurrence are important aspects to consider for patients being administered chemotherapy for gastrointestinal malignancies. In this study on patients with gastrointestinal tumors who were administered platinum-containing chemotherapy, we retrospectively analyzed the influencing factors of CIT after chemotherapy to provide a basis for preventing and reducing the occurrence of CIT in such patients and improving their quality of life.

## Materials and Methods

### Subjects and Study Variables

Subjects: We included 750 patients who were pathologically diagnosed as having digestive system malignancies and were treated with oxaliplatin-containing regimens (XELOX, FOLFOX, or SOX) in the First Hospital of Jilin University from January 2019 to July 2020. (2) Study variables: We collected clinical and demographic data for the following aspects: gender, age, BMI, site of primary tumor, degree of differentiation, chemotherapy regimen, course of treatment, total dose of oxaliplatin, the total doses of S-1, capecitabine or 5-FU, tumor (T), lymph node metastasis (N), distant metastases (M), hepatic metastasis (present/absent), lung metastasis (present/absent), other metastasis (present/absent), gene mutation, radiotherapy, aspartate transferase (AST), aminotransferase (ALT), albumin, total bilirubin (TBil), blood urea nitrogen, serum creatinine, total cholesterol (TC), triglyceride (TG) levels, low-density lipoprotein levels (LDL), high-density lipoprotein (HDL) levels, blood glucose levels, white blood cell count, neutrophil count, monocyte count, lymphocyte count, basophil count, eosinophil count, hemoglobin levels, baseline thrombocyte count, lymphocyte-to-monocyte ratio (LMR), neutrophil-to-lymphocyte ratio (NLR), platelet-to-lymphocyte ratio (PLR), transferrin, C-reactive protein levels (CRP), total iron-binding capacity, ferritin levels, serum iron levels, serum d-dimer level, CD3+, CD3+CD4+, CD4+CD8+, CD3+CD8+, CD4/CD8, natural killer cells (NK cells; CD16+56+), CD19+, whole body phase angle, and skeletal muscle mass index (SMI).

### Inclusion Criteria

(1) Patients aged ≥ 18 years; (2) patients with pathologically confirmed gastrointestinal malignancies; (3) patients receiving postoperative adjuvant or advanced first-line chemotherapy, specifically XELOX, SOX, and FOLFOX platinum-containing regimens; and (4) patients who showed the lowest platelet count of < 100 × 10^9^/L for the first time during chemotherapy.

### Exclusion Criteria

(1) Patients with thrombocytopenia caused by other reasons, such as hypersplenism, hematological diseases, and bone marrow metastasis; (2) patients having co-existing primary malignant tumors of other systems; and (3) patients given hormones and other drugs that affect the platelet count besides the chemotherapy regimen. (4) During treatment, subsequent treatment was intolerable due to adverse reactions.

### Definition

(1) CIT was defined as the platelet count in peripheral blood being < 100 × 109/L [6]. The observation index of this study is the first occurrence of CIT during treatment and does not involve whether CIT occurs again in the subsequent period. For patients who did not develop CIT, the study cutoff time was the end of treatment or progression. (2) It should be noted that although most studies have identified *P* < 0.05 was defined as statistically significant. In this study, however, baseline variables that were clinically relevant or had a univariate relationship with outcome and the number of available events were taken into account. Finally, univariate analysis of *P*-value threshold < 0.1 was listed as meaningful, and then, multivariate binary logistic regression analysis was performed (*P* < 0.05).

### Statistical Analysis and Detection Method

A database was established using the SPSS 25.0 statistical software; measurement data were analyzed using the *t* test, and the *χ*^2^ test was used for count data. The influencing factors that may cause CIT were included in the univariate analysis; the factors identified as significant by univariate analysis (*P* < 0.10) were subjected to multivariate analysis, which used logistic regression to screen out the independent risk factors of CIT (*P* < 0.05). Based on the independent influencing factors to obtain the predicted probability, the area under the receiver operating characteristic (ROC) curve was separately calculated, and the optimal cutoff value was finally determined.

Lymphocyte subsets include CD3+, CD3+CD4+, CD4+CD8+, CD3+CD8+, CD4/CD8, natural killer cells (NK cells; CD16+56+), and CD19+; the measurement method is mainly flow cytometry, and the unit is percentage.

## Results

In this study, we included a total of 750 patients who underwent gastrointestinal chemotherapy (Table 1): 223 in the CIT group and 527 in the control group. The CIT group had 81 women and 142 men, and 98 patients were younger than 60 years and the remaining were older than 60 years. The non-CIT group had 332 men and 195 women, and 278 patients were younger than 60 years and the remaining were older than 60 years. The mean age of patients was 60.26 ± 8.72 years in the CIT group and 57.21 ± 10.36 years in the non-CIT group.

**Table 1:** Patient characteristics

	Non-CIT group	CIT group
Gender		
Men	332	142
Women	195	81
Age (years)		
< 60	98	278
≥ 60	125	249
Site of primary tumor		
Stomach	186	84
Right colon	82	35
Left colon	124	49
Rectum	135	55
T^a^ stage		
T1	19	5
T2	22	12
T3	329	137
T4	119	57
N^b^ stage		
N0	31	105
N1	85	195
N2	52	133
N3	42	66
M^c^stage		
M0	154	405
M1	67	120

^a^Tumor (T)

^b^Lymph node metastasis (N)

^c^Distant metastases (M)

### Collinearity Test

We conducted a collinearity test on all included variables and found that there was collinearity between TC and TG, HDL, LDL. There is collinearity between white blood cell count, NLR, and PLR and neutrophil count, monocyte count, lymphocyte count, and baseline thrombocyte count. There is collinearity between CD3+, and CD3+CD4+, CD4+CD8+, and CD3+CD8+. Based on clinical considerations, we finally eliminated TC, white blood cells, NLR, PLR, and CD3+ which did not perform single-factor analysis.

### Univariate Analysis

Depending on whether or not CIT occurred, we divided the patients into two groups: the CIT group and the non-CIT group. Univariate analysis was carried out on the general data of the two groups, and the results (Table 2) revealed that 17 factors were closely related to the occurrence of CIT, namely age, lymph node metastasis (N) stage, metastasis (M) stage, lung metastasis, other site metastasis, chemotherapy regimen, course of treatment, total dose of oxaliplatin, AST, albumin, neutrophils, monocytes, baseline platelets, transferrin, NK cells, phase angle, and SMI (*P* < 0.10).

**Table 2:** Single-factor analysis of CIT^a^

Variable	Non-CIT group	CIT group	*χ*^2^	*P*
Gender	527	223	0.031	0.860
Age (years)			4.860	0.027
< 60	278	98		
≥ 60	249	125		
Site of primary tumor	527	223	0.468	0.926
T^b^ stage	527	223	1.731	0.630
N^c^ stage	527	223	7.766	0.051
M^d^ stage	527	223	4.608	0.032
Degree of differentiation	527	223	0.934	0.920
Chemotherapy regimen	527	223	9.932	0.042
Course of treatment	527	223	80.065	< 0.01
The total dose of oxaliplatin (mg)	905.15 ± 370.47	671.67 ± 334.33		< 0.01
The total dose of 5-fluorouracil (mg)	168.0 (13.44–252.0)	103.2 (8.4–196.0)		0.030
Lung metastasis			3.917	0.048
Yes	20	16		
No	507	207		
Other site metastasis			16.451	0.036
Yes	198	25		
No	476	51		
Gene mutation	527	223	1.766	0.184
Radiotherapy	527	223	0.548	0.459
AST^e^ (U/L)	21.52 ± 12.20	23.56 ± 13.47		0.043
ALT^f^	20.08 ± 13.91	21.79 ± 16.24		0.172
Albumin (g/L)	39.56 ± 4.36	37.64 ± 4.14		< 0.01
TBil^g^	12.98 ± 7.44	12.72 ± 5.24		0.590
Blood urea nitrogen	5.10 ± 1.71	5.36 ± 4.08		0.346
Serum creatinine	66.87 ± 16.25	67.93 ± 23.20		0.538
TG^h^	1.49 ± 0.78	1.58 ± 0.96		0.210
HDL^i^	1.19 ± 0.47	1.14 ± 0.24		0.196
LDL^j^	2.91 ± 0.86	2.94 ± 0.84		0.721
Blood glucose levels	5.48 ± 1.60	5.60 ± 1.57		0.350
Lymphocyte count	1.77 ± 0.56	1.70 ± 0.53		0.138
Monocyte count (× 10^9^/L)	0.38 ± 0.16	0.34 ± 0.12		< 0.01
Neutrophil count (× 10^9^/L)	3.19 ± 1.60	2.90 ± 1.23		0.008
Eosinophil count	0.19 ± 0.17	0.18 ± 0.15		0.494
Basophil count	0.03 (0.02–0.04)	0.02 (0.02–0.04)		0.101
Hemoglobin levels	126.12 ± 20.61	127.34 ± 21.25		0.461
Baseline thrombocyte count (× 10^9^/L)	255.54 ± 85.83	213.96 ± 71.06		< 0.01
CPR^k^	3.02 (2.27–6.62)	3.02 (2.49–6.53)		0.770
Transferrin (g/L)	2.42 (2.00–2.83)	2.28 (2.03–2.64)		0.059
Serum iron levels	11.05 ± 5.67	11.46 ± 5.85		0.488
Total iron-binding capacity	57.52 ± 43.04	52.11 ± 9.83		0.154
Ferritin levels	98.75 (29.40–220.93)	99.10 (42.85–234.60)		0.440
D-dimer level	0.85 (0.51–1.40)	0.81 (0.48–1.78)		0.849
CD3+CD4+	40.29 ± 8.48	39.44 ± 9.08		0.320
CD3+CD8+	27.18 ± 9.16	27.30 ± 10.14		0.897
CD4+CD8+	0.73 (0.43–1.25)	0.68 (0.43–1.21)		0.640
CD4/CD8	1.73 ± 0.95	1.71 ± 0.89		0.859
CD19+	9.09 ± 4.08	8.79 ± 4.52		0.484
NK^l^ cell (%)	19.78 ± 11.33	22.28 ± 20.10		0.071
Whole body phase angle (°)	4.93 ± 0.78	5.07 ± 0.72		0.080
SMI^m^ (%)	9.34 ± 1.77	9.70 ± 2.09		0.056
BMI	22.53 ± 3.28	22.52 ± 2.84		0.966

^a^Chemotherapy-induced thrombocytopenia (CIT)

^b^Tumor (T)

^c^Lymph node metastasis (N)

^d^Distant metastases (M)

^e^Aspartate transferase (AST)

^f^Aminotransferase (ALT)

^g^Total bilirubin (TBil)

^h^Triglyceride (TG)

^i^High-density lipoprotein (HDL)

^j^Low-density lipoprotein (LDL)

^k^C-reactive protein levels (CRP)

^l^Natural killer cells (NK cells)

^m^Skeletal muscle mass index(SMI)

### Multivariate Analysis

The abovementioned 17 factors were introduced into the binary logistic multivariate regression model, and forward LR was chosen. Finally, the following five statistically significant factors were identified, namely the M stage, total dose of oxaliplatin, albumin, baseline thrombocyte count, and NK cell; these were independent risk factors for developing CIT (*P* < 0.05), as shown in Table 3.

**Table 3:** Multivariate logistic regression analysis of CIT^a^

	B	S.E.	Wald	*P* value	Exp (B)
M^b^ stage	− 1.019	0.407	6.266	0.012	0.361
Total dose of oxaliplatin (mg)	− 0.002	0.001	11.282	0.001	0.998
Albumin (g/L)	− 0.119	0.047	6.297	0.012	0.888
Baseline thrombocyte count (× 10^9^/L)	− 0.015	0.003	22.742	< 0.01	0.985
NK^c^ cell (%)	0.018	0.008	5.374	0.020	1.019

^a^Chemotherapy-induced thrombocytopenia (CIT)

^b^Distant metastases (M)

^c^Natural killer cells (NK cells)

### Nomogram and Calibration Curve

**Fig. 1:**
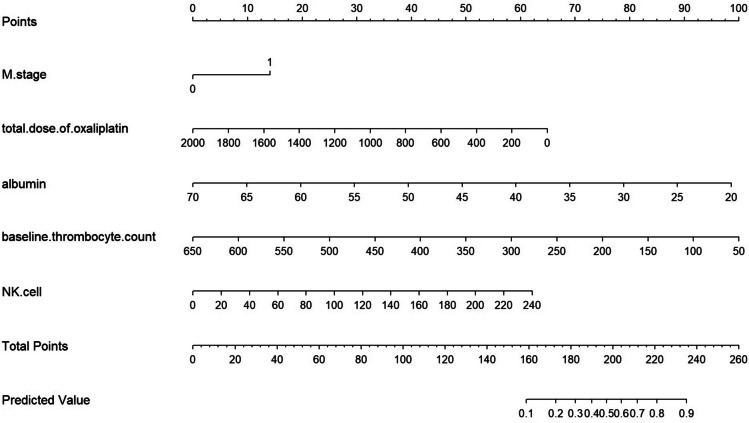
Nomogram

**Fig. 2:**
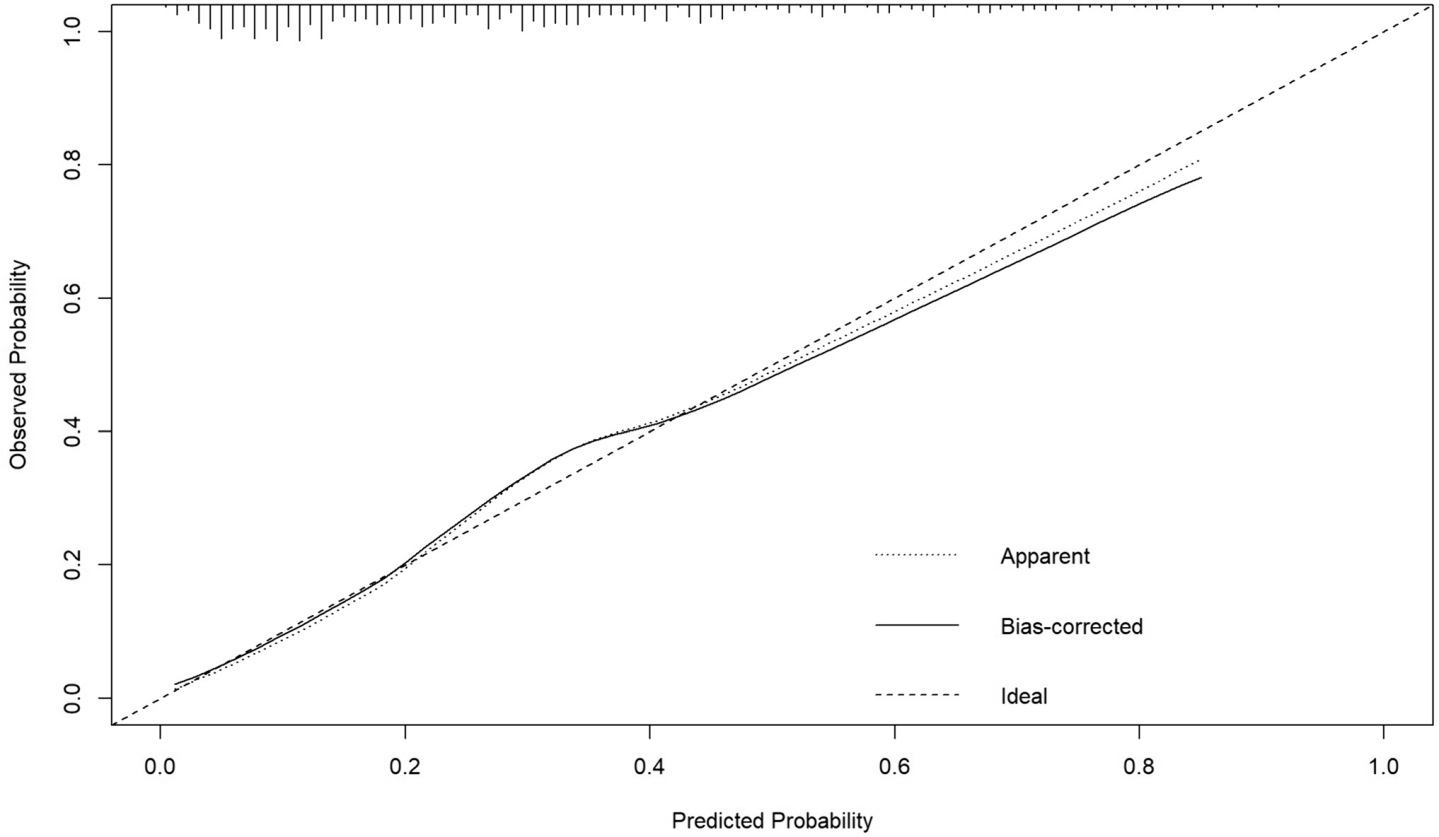
Calibration curve. Illustrate: The horizontal axis represents the predicted incidence probability, and the vertical axis represents the actual incidence probability. The diagonal dashed line represents the perfect prediction of the ideal model. If the trends of the simulated curve and the actual curve are more consistent, the fit with the diagonal dashed line would be better, thus indicating a better prediction performance of the model

Based on the results of multivariate logistic regression analysis, R software was used to establish a graph prediction model of thrombocytopenia associated with chemotherapy containing oxaliplatin in patients with gastrointestinal tumors, as shown in Fig. 1. The calibration curve (Fig. 2) was drawn according to the predicted value of the multivariate logistic regression analysis to analyze the fitting degree between the predicted situation and the actual situation. The calibration curve results show that the trajectory of the simulated curve and the actual curve is basically the same, which indicates that the model has a strong consistency.

### The Predicted Probability and ROC Curve

**Fig. 3:**
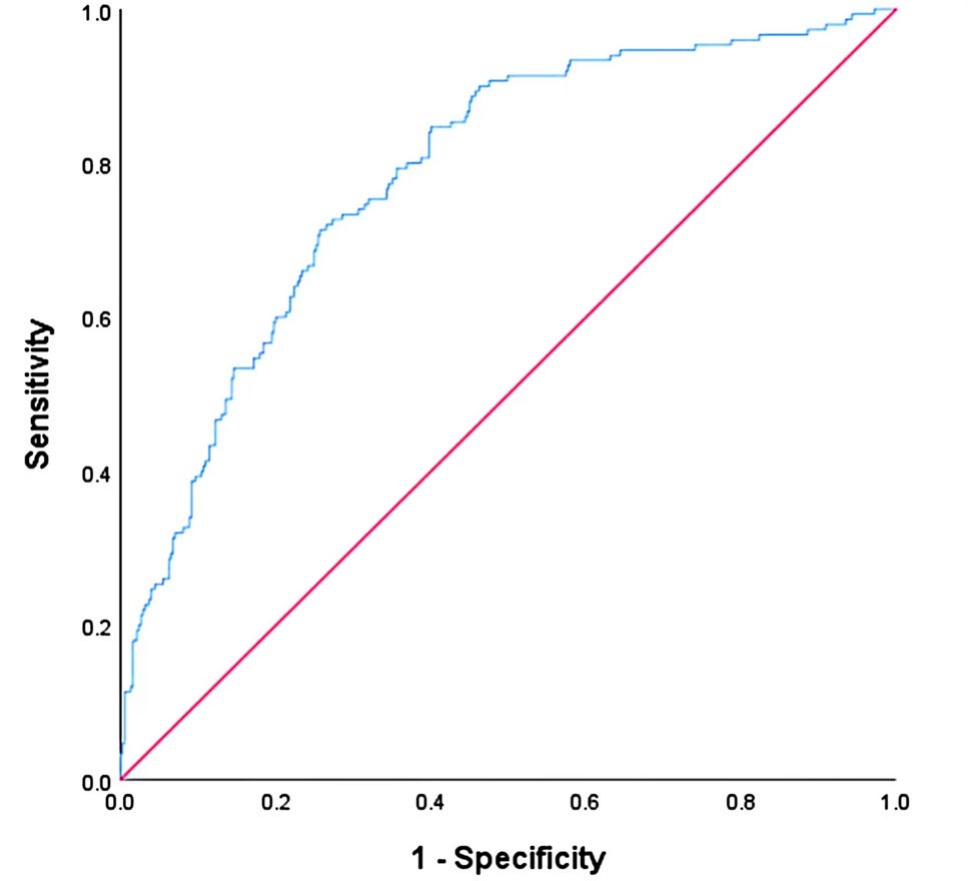
ROC curve

After binary logistic multivariate regression analysis, the predicted probability was obtained. Figure 3 shows the ROC curves of the predicted probability which presented by the blue line closest to the upper left corner with the largest area under the curve (0.877). The areas under the curve for the M stage, total dose of oxaliplatin, albumin, baseline thrombocyte count, and NK cell are 0.547, 0.308, 0.376, 0.268, and 0.555, respectively. The best cutoff value of the area under the curve of the predicted probability was 0.3579613, with a sensitivity of 78.9% and a specificity of 81.8%.

## Discussion

Hematological toxicity, particularly CIT, is a major dose-related adverse effect of many cytotoxic chemotherapeutics. CIT often increases bleeding, delays chemotherapy, and limits the dose of chemotherapeutics, consequently reducing the efficacy of chemotherapy, affecting the quality of life of patients, and increasing mortality and medical costs. Therefore, assessing the risk of CIT in patients with gastrointestinal malignancies undergoing chemotherapy is of great clinical significance.

Studies have shown that women are less likely to suffer from low platelets due to their high basal platelet count [9–12]. However, in a pooled analysis of five randomized trials, Abdel-Rahman demonstrated that not all grades of thrombocytopenia significantly differed between women and men [13]. In our study, thrombocytopenia incidence did not significantly differ between men and women. We speculate that it may be because male and female patients were not stratified for analysis. In subsequent studies, we will stratify male and female patients to separately analyze its relationship with CIT.

Using multivariate analysis, Yamada [11] found thrombocytopenia to be more frequent in patients aged > 70 years after CS treatment. Similarly, studies have reported that with increasing age, the hematopoietic function of bone marrow slows down and is more vulnerable to myelosuppression during chemotherapy [14–16]. Conversely, Professor Yo Han showed that thrombocytopenia incidence did not significantly differ between different age groups (6.4% over 65 years *vs*. 3.9% under 65 years) [17]. Our study only found that age is an influencing factor of CIT in univariate analysis; we can combine multiple indicators to further analyze their relationship with CIT in future.

Some studies have pointed out that patients with a lower body mass index (BMI), moderate/severe malnutrition, and an Eastern Cooperative Oncology Group (ECOG) score of 1 are more likely to suffer from thrombocytopenia [18–20]. Our study included nutrition-related indicators such as serum albumin, phase angle, and SMI. The results showed that serum albumin is an independent risk factor for CIT, while phase angle and SMI were only found to affect platelets in univariate analysis. But this provides us with ideas for subsequent research, whether it is possible to include more body composition analysis indicators, conduct combined analysis of different indicators, or calculate unit area to further study the relationship with CIT.

Woo et al. [21] reported the incidence of thrombocytopenia due to myelosuppression as 45–77%. Platelet count reduces below 75 × 10^9^/L usually after 10 days of oxaliplatin administration, with few symptoms of thrombocytopenia, such as congestion, purpura, or mucosal bleeding. Studies have suggested that oxaliplatin causes CIT mainly through the following three mechanisms [22]: (1) oxaliplatin has a myelosuppressive effect, and thus, it reduces the activity and function of hematopoietic stem cells in the bone marrow, resulting in decreased platelet production; (2) repeated oxaliplatin treatment induces and maintains immune responses, leading to immune thrombocytopenia [23]; and (3) oxaliplatin causes liver sinusoidal injury, leading to portal hypertension, splenomegaly, and hypersplenism, which reduce the platelet count. The main mechanism of oxaliplatin-induced thrombocytopenia is myelosuppression, whereas the incidence of splenomegaly and immune response mechanisms is relatively low in clinical practice. Studies have shown that the incidence of CIT increases significantly with the increase in chemotherapy cycles. An observational study of 320 patients with CIT published in 2011 showed that the incidence of CIT was 0.16%, 17.8%, 37.8%, and 43.8% in the first, second, third, and fourth cycles of chemotherapy, respectively [24]. Some studies have shown that the number of chemotherapy cycles [median (25%, 75%)] at the first occurrence of CIT in patients receiving a platinum-containing regimen were 3 and the cumulative dose of oxaliplatin received at the time of the first occurrence of CIT was 569.7 mg ± 273.4 mg [25]. We found that total oxaliplatin dose was an independent risk factor for CIT, consistent with the above studies. In the future, we will include indicators such as body surface area to further analyze the relationship between unit oxaliplatin dose and CIT.

Other studies have shown that different races have different adverse reactions to capecitabine. For example, the incidence of III–IV CIT was significantly higher in Chinese patients at a dose intensity of 900 mg/m^2^ (9%; overall CIT incidence, 84%) than in the Western population at a dose intensity of 1000 mg/m^2^ (2.1%; overall CIT incidence, 49%), suggesting that capecitabine has a greater effect in Asian populations [26]. In our study, patients with gastric cancer were treated with the SOX regimen, and patients with colorectal cancer were treated with the XELOX or FOLFOX regimen. By stratifying different types of gastrointestinal cancers and different chemotherapy regimens, the total doses of S-1, capecitabine, and 5-FU were calculated, and then their correlation with CIT was analyzed; our study did not conclude that total fluorouracil dose was an independent risk factor for CIT. In combination with clinical practice, it is considered that it may be due to the different metabolic pathways and mechanisms of action of fluorouracil and oxaliplatin, fluorouracil belongs to the antipyrimidine antimetabolite, which inhibits deoxythymididate synthase by converting to 5-fluorouracil deoxynucleotide in vivo, blocking the conversion of deoxyuriglycate to deoxythymidylate and inhibiting DNA synthesis. It may cause a heavier myelosuppressive response, mainly manifested by varying degrees of granulocytopenia, which occurs earlier but relatively rarely [27].

The rate of depletion of hematopoietic cells in the bone marrow depends on the half-life of the cells. The half-lives of white blood cells, platelets, and red blood cells were 6 h, 5–7 days, and 120 days, respectively, indicating that platelets were more easily affected and decreased. The low platelet level before treatment sensitively reflects compromised hematopoietic function of bone marrow granulosa system. Clinically, such patients are more prone to myelosuppression after chemotherapy. Our current findings reveal that the baseline platelet count is an independent risk factor for CIT, emphasizing the significance of a low baseline platelet count before treatment in predicting CIT occurrence. Combined with the situation of some studies, the occurrence of thrombocytopenia before treatment can be attributed to the following: (1) bone metastases occur in the late stage of the tumor, and tumor cells have spread to the marrow hematopoietic system, thus directly inhibiting the normal hematopoietic function of the bone marrow [28]; (2) poor eating and malnutrition in patients with gastrointestinal tumors affect bone marrow hematopoiesis. Therefore, pretreatment of patients with low baseline platelets via prophylactic use of thrombopoietin and improved nutrition nutritional status is highly significant for clinical treatment.

Thrombopoietin (TPO) is an essential factor for megakaryocyte growth and is the only important regulator of platelet production. Endogenous TPO is mainly produced by the liver (70%) and kidney. In patients with liver metastasis, the liver structure is affected, and there are notable pathological changes in the normal liver tissue around the lesion; this further decreases chemotherapy tolerance of the liver [29]. The oxaliplatin-containing chemotherapy regimen leads to liver function damage and causes spleen enlargement, which is a potential cause of persistent thrombocytopenia after oxaliplatin chemotherapy [30]. Studies have shown that the cumulative incidence of thrombocytopenia as an adverse event was 22% (41/184), 24% (181/752), and 23% (32/142) in patients with only liver metastases, liver metastases combined with other metastases, and without liver metastases, respectively, and the difference in these incidence rates was not statistically significant [31]. Our study found that lung metastases distant metastases to other sites besides the liver and lung seemed to affect platelet counts after chemotherapy only in univariate analysis. We herein could not identify whether liver metastases can affect the generation of endogenous TPO and consequently the platelet count and thus may need to be further explored in clinical practice in the future.

Our study identified NK cell as an independent risk factor for CIT; however, the predictive value of NK cell for inducing CIT remains unknown. Clinically, albumin indicators can roughly reflect the patient’s nutritional status, and NK cells can also reflect the patient’s immune function to a certain extent. The nutritional status of patients may affect their immune function, so NK cells may be related to CIT, but this conclusion may require further clinical research and analysis. Unfortunately, the area under the ROC curve for either factor was small, and the predictive value was low. Given the high reliability of joint prediction, we can carry out joint factor analysis for patients with gastrointestinal tumors in the clinic to intervene and treat patients who are prone to CIT and improve the outcomes of these patients.

Thus far, few studies have established CIT risk prediction models based on clinical and laboratory indicators. In our study, the best cutoff value for the area under the curve of the predicted probability was 0.3579613 (sensitivity, 78.9%; specificity, 81.8%). These differences may be attributed to a number of different factors, such as the number of patients, the definition of CIT, and differences in the chemotherapy agent administered.

The total dose of oxaliplatin, M stage, albumin, baseline platelet count, and NK cell was independent risk factors for CIT. The sequentially constructed histogram model had a good predictive effect on the risk of thrombocytopenia caused by oxaliplatin chemotherapy in patients with gastrointestinal malignancies.

The present study had several limitations. The selection of subjects was from a single center, and the variety of potential predictors collected was limited by clinical practice. Furthermore, this study was a retrospective study with limited collection of clinical data. The conclusions obtained in this study need to be further verified in multicenter studies and clinical practice. In a future study, we hope to increase the sample size and include other variables in clinical practice to identify the patients at risk of CIT with greater accuracy, guide clinical practice, and improve the treatment effect.

## Conclusion

Taken together, based on readily available and easily measurable indicators, the M stage, total dose of oxaliplatin, albumin, the baseline platelet level, and NK cell were identified to be associated with CIT in the multivariate model. The sequentially constructed histogram model had a good predictive effect on the risk of thrombocytopenia caused by oxaliplatin chemotherapy in patients with gastrointestinal malignancies. The predicted probability could help preemptively identify high-risk patients for chemotherapy-induced thrombocytopenia. Although this model may help identify patients at risk for CIT, more and larger multicenter studies are needed before clinical application.

## Data Availability

Materials described in the manuscript, including all relevant raw data, will be freely available to any scientist wishing to use them for non-commercial purposes, without breaching participant confidentiality.
